# THE LONGITUDINAL INFLUENCE OF MENTAL DEMANDS OF OCCUPATION ON COGNITIVE FUNCTION AMONG MEXICAN OLDER ADULTS

**DOI:** 10.1093/geroni/igae098.0170

**Published:** 2024-12-31

**Authors:** Mariela Gutierrez

**Affiliations:** The University of Texas Medical Branch, Texas City, Texas, United States

## Abstract

This study addresses the role of occupation as a determinant of cognitive health in low-middle income countries (LMICs), focusing on Mexico. While high-income countries have established links between cognitively stimulating jobs and preserved cognitive functioning, research in LMICs is limited. The aim is to investigate the association between lifetime mental work demands and memory changes from 2001 to 2018 in Mexican older adults. We used data from the Mexican Health and Aging Study (MHAS), a nationally representative study of individuals aged 50 and above. Participants main lifetime occupation is classified according to the Mexican Classification of Occupations. MHAS data was linked to the Occupational Information Network (O*NET), which provides descriptors for various job domains, including cognitive abilities. A mental demand index was created from ten O*NET items, and a memory score was derived from immediate and delayed recall of an 8-word list. Linear mixed effect models with random intercepts were conducted to analyze the association between mental demands of work and memory scores, adjusting for demographic, health, and job-related variables. While the association between mental work demands and memory changes was not statistically significant, estimates indicate a decline in memory scores over time. The findings reveal more memory decline among agricultural and domestic/service workers compared to professionals and administrative workers. Other work factors associated declines in memory are receiving benefits from work, age at which individuals began their work. This research contributes to understanding the complex interplay between occupation, cognitive health, and aging in LMICs, particularly in the Mexican context.

